# Confluences of exceptional points and a systematic classification of quantum catastrophes

**DOI:** 10.1038/s41598-022-07345-7

**Published:** 2022-03-01

**Authors:** Miloslav Znojil

**Affiliations:** 1 The Czech Academy of Sciences, Nuclear Physics Institute, Hlavní 130, 250 68 Řež, Czech Republic; 2 Department of Physics, Faculty of Science, University of Hradec Králové, Rokitanského 62, 50003 Hradec Králové, Czech Republic; 3 Institute of System Science, Durban University of Technology, P. O. Box 1334, Durban, 4000 South Africa

**Keywords:** Quantum physics, Applied mathematics, Pure mathematics

## Abstract

In the problem of classification of the parameter-controlled quantum phase transitions, attention is turned from the conventional manipulations with the energy-level mergers at exceptional points to the control of mergers of the exceptional points themselves. What is obtained is an exhaustive classification which characterizes every phase transition by the algebraic and geometric multiplicity of the underlying confluent exceptional point. Typical qualitative characteristics of non-equivalent phase transitions are illustrated via a few elementary toy models.

## Introduction

In the conventional descriptions of unitary evolution of quantum systems in Schrödinger picture (SP^1^) the information about dynamics is all carried by the Hamiltonian. The predictions of experiments are all based on the solution of Schrödinger equation

1 $$\begin{aligned} \mathrm{i}\frac{d}{d t}|\psi (t)\rangle =H\, |\psi (t)\rangle \, ,\ \ \ \ \ \ \ |\psi (t)\rangle \in \mathcal{V}\,. \end{aligned}$$

A broader perception of the concept of quantum dynamics will be advocated here, with emphasis upon *qualitative * aspects of the role of parameters.

Among several sources of inspiration of our project the most obvious one is the Thom’s theory of catastrophes^2,3^. As long as such a theory is of a purely geometric nature^4^, it is applicable, predominantly, to the classical dynamical systems. In this domain it offers, first of all, a systematic classification of structures and of the possible changes of structures of the long-term classical equilibria^5^. The applicability and/or an immediate transfer of these ideas to quantum systems are limited^6–10^.

According to papers^11–17^, new eligible directions of research emerged after a turn of attention to the Kato’s concept of exceptional point (EP^18^). After a small change $g \rightarrow g^{(EP)}$ of a real or complex parameter in Hamiltonian $H=H(g)$, such an operator ceases to be diagonalizable. Hence, the bifurcation of the Thom’s classical equilibria can find its genuine quantum analogue in the passage of the parameter through its real or complex value $g^{(EP)}$. The latter possibility is, in essence, also the key point of our present paper. In our text we will try to develop the idea in a certain more systematic and constructive manner.

A decisive key to the realizability of the project can be seen in the Bender’s and Boettcher’s change of the traditional paradigms^19^. Indeed, it was them who conjectured that the unitary evolution could be, under certain conditions, realized and described even when the generator *H* of evolution of the wave function in Schrödinger Eq. (1) becomes, in an apparent contradiction to the well known Stone theorem^20^, manifestly non-Hermitian. And precisely this change of paradigm (cf. also the detailed outline of the resulting consistent quantum theory of closed systems as reviewed, more than ten years ago, in papers^21,22^) opened also the way towards the change of the status of the notion of EPs from a strictly mathematical tool as developed in the Kato’s book to one of the most important concepts in experimental physics—see, e.g., paper^23^ outlining the related “roadmap for future studies and potential applications”.

The core of our present message will lie in a combination of the purposeful theoretical use of parameter-dependent non-Hermitian Hamiltonians with a detailed analysis of some of the consequences in the ambitious phenomenological context of description and classification of a broad class of phenomena called quantum phase transitions^24^. Naturally, the feasibility of our project will require a certain methodically motivated narrowing of its scope. Thus, in contrast to the more conventional perception of the quantum phase transition phenomena as described in textbook^24^ (and, typically, associated with the spontaneous symmetry breaking), our present approach to the problem of phases will be slightly different, more closely associated with the potential passage of the quantum system in question strictly through its EP singularity.

In the context of such a theory (rendered consistent by the non-Hermiticity of *H*) we will only consider a number of elementary toy models, with emphasis upon the quick, non-numerical solvability of the related Schrödinger Eq. (1). It is worth adding that in such a case (to be related here, for the sake of simplicity, just to the spectral phase transitions) one of the phases may (though need not) be ill-defined in a way depending on the respective presence or absence of the complexification of the spectrum near the EP (see, e.g.,^25^ for a few illustrative examples of the latter, slightly less well known possibility of having no complexification).

In the currently highly popular pragmatic context of the phenomenological applicabilty and of the proposals and predictions of the results of experiments, the unavoidable methodical limitations of our present considerations using oversimplified toy models will be even more visible and restrictive. In this respect the readers may be recommended to fill the experiment-related gaps in our text by following the currently existing and rich specialized literature (see, e.g., the freshmost reviews of non-Hermitian physics in^26,27^).

In the latter frame we will only emphasize the central role played, in the underlying mathematics and physics, by the ubiquitous^12^ Kato’s notion of EPs. In the language of mathematics we only intend to complement the contemporary popular but rather formal reference to EPs in various realistic models by a slightly more ambitious theoretical interpretation of the EP concept referring to its non-equivalent realizations.

## Unitarity-of-evolution constraint

Among the existing applications of qualitative considerations to quantum dynamics we felt particularly addressed by the mathematical studies in which the EP limits were of order two (EP2). In this scenario, just some two neighboring eigenvalues $E_n(g)$ and $E_{n+1}(g)$ of *H*(*g*) are assumed to merge and complexify at $g = g^{(EP)}= g^{(EP2)}$.

The latter studies were often motivated by the physics of systems exhibiting a genuine quantum phase transition^28,29^. According to our most recent commentary^30^, most of these systems have been considered “open”, interacting with a certain not too well specified “environment”. As a consequence, the bound states remained unstable, with the energies which need not be kept real^31^. In such an open-system setup a typical Hamiltonian *H*(*g*) is non-Hermitian so that its EP singularities of the $N$-th-order may be complex, $g= g^{(EPN)} \in {\mathbb {C}}$. One can, nevertheless, hardly speak about fundamental theory because the “input” information about the open-system dynamics (and, in particular, about the environment) remains incomplete.

Our present attention will be restricted to the closed systems characterized by the unitarity of their evolution. One of the key technical consequences is that the postulate of unitarity must be, due to the Stone theorem^20^, necessarily connected with the postulate of Hermiticity of the Hamiltonian.

The way out of the apparent impasse has only been discovered very recently. It appeared sufficient to replace the conventional textbook SP paradigm by its straightforward upgrade which works with the *two * non-equivalent Hermitian conjugations and which may be called pseudo-Hermitian quantum mechanics (PHQM, see its review^22^).

### Quantum observables in pseudo-Hermitian representation

In the PHQM SP approach the EP singularity may mark a natural boundary of the formal acceptability of any candidate for quantum Hamiltonian. The theory emphasizes that the mere specification of the linear space $\mathcal{V}$ and the related knowledge of the ket-vector solutions $|\psi (t)\rangle \in \mathcal{V}$ of Schrödinger Eq. (1) are insufficient. What is considered equally important is the freedom of the choice of the physical inner product between states. This is equivalent to the specification of a correct dual space $\mathcal{V}'$ of the linear functionals in $\mathcal{V}$. Such a choice is known to be ambiguous (see, e.g., p. 246 in^1^). Still, in contrast to the widespread beliefs, this ambiguity may be useful, bringing several immediate theoretical challenges as well as practical benefits^32^.

Some of the subtler aspects of the problem did not find their ultimate clarification yet^33^. Nevertheless, under certain additional technical assumptions the apparent paradox has already been resolved, almost thirty years ago, in review paper^34^. The ambiguity of the abstract theory, i.e., the ambiguity of the choice of the correct physical Hilbert space $\mathcal{H}_{\mathrm{(physical)}}=[\mathcal{V},\mathcal{V}'_{\mathrm{(physical)}}]$ has been shown removable. It has been explained that there exists a very natural method of the necessary unique specification of the correct antilinear duality map $\mathcal{T}_{\mathrm{(physical)}}:\,\mathcal{V}\ \rightarrow \ \mathcal{V}'_{\mathrm{(physical)}}$.

In the literature one still encounters a few obstinate terminological misunderstandings. One of their sources lies in the fact that the operator $\mathcal{T}_{\mathrm{(physical)}}$ of the correct Hermitian conjugation need not necessarily have an easily obtainable realization (cf., e.g.,^35–38^). The reconstruction of the physical inner-product space $\mathcal{H}_{\mathrm{(physical)}}$ is, therefore, most often postponed till the very end of the calculations. Temporarily, the correct physical space is being replaced by its simplified, user-friendlier alternative $\mathcal{H}_{\mathrm{(auxiliary)}}$. In spite of being manifestly unphysical, the key advantage of the latter choice lies in the simplification of the conjugation. Its most straightforward form $\mathcal{T}_{\mathrm{(auxiliary)}}:\,\mathcal{V}\ \rightarrow \ \mathcal{V}'_{\mathrm{(auxiliary)}}$ is realized as the action which transforms the column-vector *alias * ket-vector $|\psi \rangle \in \mathcal{V}$ into its conventional “Dirac’s” conjugate of textbooks, i.e., into its bra-vector partner $\langle \psi | \in \mathcal{V}'_{\mathrm{(auxiliary)}}$ which is constructed as a row-vector composed of the complex-conjugate elements of its partner $|\psi \rangle$.

For the users of the PHQM SP theory it is sufficient to know that the decisive simplification of it applications is achieved via a consequent representation of all of the states in $\mathcal{H}_{(auxiliary)}$ rather than in $\mathcal{H}_{(physical)}$. The *only * space in which one performs calculations is $\mathcal{H}_{(auxiliary)}$. Hence, the use of the Dirac’s bra-ket notation conventions cannot lead to any contradictions. Under this convention it is easy to evaluate any correct inner product $(\psi _1,\psi _2)_{(physical)}$ in $\mathcal{H}_{(physical)}$ in terms of its unphysical partner in $\mathcal{H}_{(auxiliary)}$ because we are allowed to abbreviate $(\psi _1,\psi _2)_{(auxiliary)}=\langle \psi _1|\psi _2\rangle$. The representation of the amended inner product $(\psi _1,\psi _2)_{(physical)}$ remains based on the definition $(\psi _1,\psi _2)_{(physical)}=\langle \psi _1|\Theta |\psi _2\rangle$ in which the new symbol $\Theta$ (called Hilbert space metric) can in fact carry a nontrivial part of the information about dynamics.

The picture of reality remains internally consistent. Whenever one considers a parameter-dependent (and, say, analytic) family of SP Hamiltonians *H*(*g*) admitting an EP singularity at a (real or complex) EP value $g=g^{(EP)}$, one has to localize the domain $\mathcal{D}_{(physical)}$ of admissible parameter(s) *g* at which the spectrum remains real and discrete, i.e., unitarity- and closed-system-compatible. The theory is completed when one specifies also the Hilbert-space metric which is, in general, $g$-dependent, $\Theta =\Theta (g)$.

The necessary *mathematical * properties of the metric operator can be found thoroughly discussed in^22,33,34^ and in^39^. Among these properties a key role is played by the ambiguity of the assignment of the metric to a preselected SP Hamiltonian symbolized, whenever needed, by the introduction of another formal parameter $c\,$ in $\Theta =\Theta (g,c)$.

Irrespectively of the latter ambiguity, *any * Hamiltonian-compatible metric will necessarily *cease to exist * in the EP limit^40^. In parallel, our operator *H*(*g*) will cease to be diagonalizable and it will lose its status of an acceptable Hamiltonian in *the same * limit of $g\rightarrow g^{(EP)}$.

### Quantum phase transitions at exceptional points

Several well known quantum effects can be connected with some EP singularities. The limit of $g\rightarrow g^{(EP)}$ implies the end (or at least interruption) of the observability of the quantum system. In such a limit, typically (cf., e.g., the schematic model in^41^), at least one pair of energy levels merges and complexifies, i.e., the system ceases to be unitary. Besides the schematic models there also exist multiple entirely realistic samples of such a phenomenon. The best known ones are encountered in relativistic quantum mechanics. The emergence of the singularity requires there an abrupt redefinition of the Hamiltonian in which one has to incorporate the new, “unfrozen” dynamical degrees of freedom.

The necessary matching of the old (= “before EP”) and new (= “after EP”) dynamics (i.e., between the respective *ad hoc * Hamiltonians) is usually performed on a pragmatic, effective-Hamiltonian basis. The realization of the transition becomes less counterintuitive when the EP-caused loss of the observability happens to involve more than two levels. One of the most characteristic illustrative examples is the well known Landau’s^42^ strongly singular harmonic oscillator with potential

2 $$\begin{aligned} V^{(HO)}(x)=x^2-g/x^2\,. \end{aligned}$$

**Figure 1:**
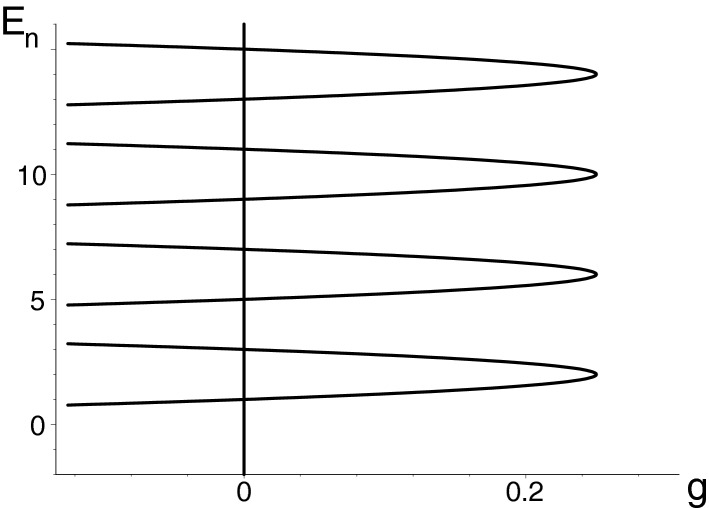
The low-lying part of the bound-state spectrum of the exactly solvable quasi-Hermitian harmonic oscillator of Eq. (2). The model possesses the confluent exceptional point tractable as the loss-of-unitarity quantum phase transition at $g=1/4$. Picture created using Maple^44,45^.

The system collapses, in suitable units, at $g=1/4$. One of the ways towards the resolution of the puzzle has been described in Ref.^43^. At $x=0$ we regularized the potential in the spirit of pseudo-Hermitian quantum theory. The collapse of the spectrum then acquired an immediate EP-related form. With the growth of attraction *g* the levels were found to merge and to form, subsequently, the complex conjugate pairs (cf. Fig. 1).

In our recent follow-up paper^46^ a closer connection has been established between the harmonic-oscillator physics of collapse and the mathematics of its exceptional points. Near $g=1/4$, in particular, explicit form has been found of all of the admissible duality maps $\mathcal{T}$ defining all of the available physical Hilbert spaces and metrics $\Theta (g,c)$. With an auxiliary regularization shift $\varepsilon >0$ of coordinates $x \rightarrow x-\mathrm{i} \varepsilon$ in (2) (which does not influence the results and can be arbitrary) the HO model has been finally shown to support the desirable degeneracies of our present interest,

3 $$\begin{aligned} \lim _{{{g}}\rightarrow {{g}}^{(EP2)}_{m}} E_{2m}({{g}})= \lim _{{{g}}\rightarrow {{g}}^{(EP2)}_{m}} E_{2m+1}({{g}})= E^{(EP2)}_m<E^{(EP2)}_{m+1} \,,\ \ \ \ m = 0,1,\ldots \,. \end{aligned}$$

From the point of view of mathematics it is utterly nontrivial that all of these exceptional-point couplings *coincide*,

4 $$\begin{aligned} {g}^{(EP2)}_{0}= {g}^{(EP2)}_{1}= {g}^{(EP2)}_{2}= \ldots = {g}^{(EP)}_{(confluent)} \, \end{aligned}$$

(see, once more, Fig. 1). By far the most interesting physics behind the latter “degeneracy of degeneracies” occurs in a small vicinity of the confluent EP value. At the slightly weaker couplings ${g} < 1/4$ the *whole * spectrum is non-degenerate and real, i.e., the system is unitary. Whenever we choose just a slightly stronger attraction ${g} > 1/4$, the reality of all of the individual energy levels gets simultaneously lost.

For a complementary, qualitatively different introductory illustration let us recall the exactly solvable square-well model $H^{(SQW)}({g})$ of Ref.^41^. The process of the loss of the observability starts there at the two lowermost bound states. With the growth of *g* (i.e., of the non-Hermiticity), the model produces an infinite sequence of the energy mergers of order two (EP2). They are real, well separated and ordered as follows,

5 $$\begin{aligned} 0<{g}^{(EP2-SQW)}_{0}< {g}^{(EP2-SQW)}_{1}< {g}^{(EP2-SQW)}_{2}< \ldots \,. \end{aligned}$$

This is a typical, generic scenario. In many other quantum models with a variable parameter (cf., e.g.,^47–49^), the EP-caused quantum-phase-transition phenomenon just exclusively involves the pairs of the merging levels. Obviously, the gradually emerging EP2s are characterized by their nonzero distance from the parametric domain $\mathcal{D}_{(physical)}$ so that they remain phenomenologically irrelevant. At the same time, their confluence as sampled by Fig. 1 has always been considered improbable and next to impossible to achieve in the laboratory^12^.

A decisive return to optimism as sampled by the theoretical results of Ref.^25^ is only of a very recent date. One has to emphasize that also in the parallel context of the possible experimental simulations the recent progress is quick. In^50^, for example, the authors argued that the models as sampled, up to some similarity transformations, in Ref.^25^ could really find an immediate experimental realization. In detail these authors have shown that the matrices controlling the evolution of the higher-order field moments of certain two-mode systems could be realizable in the zero-dimensional bosonic anti-$\mathcal{PT}$-symmetric dimers.

### The mergers of the mergers

In our present paper we will ignore the isolated EP2 mergers of a single pair of energies

$$\begin{aligned} \lim _{{{g}}\rightarrow {{g}}^{(EP2)}} [E_{j}({{g}})- E_{j+1}({{g}})]=0 \end{aligned}$$

occurring at a single excitation *j* as not too interesting. Our search will be redirected to the models exhibiting certain “mergers of the mergers”. More explicitly, we will introduce at least one other variable parameter (say, *p*) and we will search for the confluence of the EP2s themselves, i.e., of ${g}^{(EP2)}_{a}(p)$ with ${g}^{(EP2)}_{b}(p)$, etc. Thus, in an “upgraded” dynamical scenario we will search, say, for four-level merger EP4 = EP2 $\oplus$ EP2 such that

$$\begin{aligned} \lim _{{{p}}\rightarrow {{p}}^{(EP4)}} [{g}^{(EP2)}_{a}(p)- {g}^{(EP2)}_{b}(p)]=0 \end{aligned}$$

etc.

In the framework of such a project, both of our previous illustrative models proved unsatisfactory. In the former, harmonic-oscillator case, the “merger of all mergers” did occur but it remained rigid, parameter-independent, i.e., not usable for any active control of dynamics. In the other, SQW model, what remained rigid was the separation of the exceptional points. The absence of any auxiliary parameter *p* did not allow us to convert at least some of the sharp inequalities into equal signs in Eq. (5).

An encouraging partial resolution of the puzzle only came with paper^25^. We managed to match there the evolution “before EP” with the evolution “after EP”. The goal has been realized via an extreme and brutal maximal fine-tuning procedure. The graduality formula (5) has been made parameter-dependent, i.e., in our present notation, $p$-dependent. Next, the $p$-supported limiting-confluence conversion of the sharp inequality signs “<” into equal signs “$=$” in  (5) has been imposed upon *all * of the separate EP2s. The EPN degeneracy involved all of the states (the number *N* of which was chosen even). The “gradual” pattern of Eq. (5) has been replaced by its “confluent” predecessor (4).

In the models of Ref.^25^ where $N=\mathrm{dim\,}\,H^{(before\, EP)}(g) =\mathrm{dim\,}\,H^{(after\, EP)}(g)$, the construction implied the complete degeneracy of the energies,

6 $$\begin{aligned} \lim _{g \rightarrow g^{(EPN)}}\,E_n(g) = \eta \,, \ \ \ \ n = 0, 1, \ldots , N - 1\,. \end{aligned}$$

The phase-transition-mediating Hamiltonians acquired, at the matching EP instant, the *same*, strongly fine-tuned canonical form of a single *N* by *N* non-diagonal Jordan-block matrix,

7 $$\begin{aligned} \lim _{g \rightarrow g^{(EP)}}\,H^{(before/after\, EP)}(g) \sim J^{({N})}(\eta )=\left[ \begin{array}{ccccc} \eta &{}1&{}0&{}\ldots &{}0 \\ {}0&{}\eta &{}1&{}\ddots &{}\vdots \\ {}0&{}0&{}\eta &{}\ddots &{}0 \\ {}\vdots &{}\ddots &{}\ddots &{}\ddots &{}1 \\ {}0&{}\ldots &{}0&{}0&{}\eta \end{array}\right] \,. \end{aligned}$$

The explicit construction of a genuine quantum energy-level-degeneracy catastrophe proved successful and involved all of the levels in the spectrum.

In technical terms, the feasibility of the construction reflected the finite-dimensional nature of Hamiltonians $H^{(before\, EP)}(g)$ and $H^{(after\, EP)}(g)$. Although the mechanisms causing the collapse remained unchanged, the specific simultaneous EPN-based phase-transition effect (6) itself has been rendered possible. Hamiltonians $H^{(before\, EP)}$ and $H^{(after\, EP)}$ were connected and matched in a strictly continuous and both-sided “fundamental-Hamiltonian” manner.

In the early applications of the non-Hermitian degeneracies (7), many of them retained their pragmatic effective-operator open-system physical background admitting a virtually arbitrary complex $\eta$. Only in a small minority of the closed-system models with strictly real spectra the authors emphasized their dynamically complete description as well as their fundamental-theory character.

In the latter context of our present exclusive interest, multiple further new questions emerged. Some of them will be re-opened and answered in what follows.

## Results

Our present project is aimed at the search for new forms of manipulation and control of important qualitative features of quantum dynamics. Our main result can be characterized as a proposal of an EP-based quantum alternative to the classical Thom’s catastrophe theory. The essence of such a classification concerning the quantum phase transitions will lie, in its present form, in the control of the EP2-related singularities and, in particular, in the control of their confluences and/or restructuralizations.

### Purpose: unitary access to EPs in closed quantum systems

In the literature, many authors (cf., e.g., Trefethen and Embree^51^ or Krejčiřík et al^52^) studied the PHQM-related quantum systems far from their EP singularities. For this reason they did not need to distinguish too carefully between the open (i.e., intrinsically non-unitary) and closed (i.e., intrinsically unitary) quantum systems. As a consequence, several interpretations of their results happened to be unclear or even, involuntarily, misleading. Typically, whenever they correctly identified “unexpectedly wild” reaction to “small” perturbations^52^, they did not emphasize that such a scenario is only encountered in the non-unitary open-quantum-system setup.

The clarification of the apparent puzzle was published in Refs.^53,54^. For the sake of clarity we picked up there just the “extreme” matrix (7) as an unperturbed operator. Then, for any perturbed Hamiltonian

8 $$\begin{aligned} H^{}(g) = J^{({N})}(\eta )+V(g) \end{aligned}$$

we showed that the class of perturbations $V(g)=\mathcal{O}(g)$ characterized as “sufficiently small” in the conventional open-system norm of the unphysical Hilbert space $\mathcal{H}_{(auxiliary)}$ has to be re-classified as unacceptable, always containing perturbations which prove unbounded when measured in the correct closed-system norm of space $\mathcal{H}_{(physical)}$.

In Ref.^53^ these observations were complemented by the consistent closed-system interpretation of the perturbed models (8) in $\mathcal{H}_{(physical)}$. We demonstrated that the standard requirement of the smallness of the norm of *V*(*g*) in $\mathcal{H}_{(physical)}$ offers a natural picture of reality in the vicinity of EP. We argued that in connection with the evolution of models (8) in $\mathcal{H}_{(physical)}$ one can localize certain non-empty corridors of unitary access to the quantum phase transition extremes at EPs.

A clear separation between the open- and closed-system theories must always be kept sufficiently well verbalized. Partially, what is to be blamed for the existing misunderstandings is the currently widely accepted terminology. Even our present conventional usage of the term “non-Hermitian” should be taken *cum grano salis*, i.e., with understanding of its true meaning. The point is that in the closed-system context our considerations will never contradict the conventional formulations of quantum mechanics. The operators of observables will always be self-adjoint. The only necessary clarification is that in the upgraded PHQM SP framework, all of the computations are realized in a manifestly unphysical Hilbert space $\mathcal{H}_{(auxiliary)}$^22,34^. The conventional and correct physical Hilbert space (say, $\mathcal{H}_{(physical)}$) remains only available indirectly, via its representation in $\mathcal{H}_{(auxiliary)}$.

One of the key technical merits of the PHQM amendment of the theory is that the latter representation of $\mathcal{H}_{(physical)}$ is mediated by the mere amendment of the inner product. The resulting re-arrangements of the usual SP model-building recipes then really work with the operators which are non-Hermitian (in $\mathcal{H}_{(auxiliary)}$).

### Tool: Schrödinger equations on discrete lattices

The main purpose of our present message is to show that the PHQM enhancement of the flexibility of the SP formalism leads, near the EP singularities, to some particularly important consequences. This will be illustrated by a few not too complicated benchmark gain + loss Hamiltonians in which we will postulate the existence of two parameters controlling the strength of the two separate, independent gain + loss subcomponents.

Via these toy models we will demonstrate that one can achieve several desirable transmutations of the EPs (i.e., of the dynamics in their vicinity) via the mere fine-tuned interference between the remote and central gain-plus-loss interactions.

For introduction let us recall the ordinary-differential-operator non-Hermitian square-well model of Ref.^41^. The unitarity (i.e., the reality of the spectrum of bound states) has only been guaranteed there in a finite interval of the strength (say, *g*) of the non-Hermiticity. The reality (i.e., observability) has been lost due to the EP-related mechanism of the merger of the ground state with the first excited state, $\lim _{g \rightarrow g^{(EP)}_0}\,[E_0(g)-E_{1}(g)]=0$.

With the further growth of the non-Hermiticity of *H*(*g*) beyond its EP value $g^{(EP)}=g^{(EP)}_0$, further mergers occurred, and all of them were followed by the complexifications of the energies of the higher and higher excited states. The process involved, gradually, the whole spectrum, resulting in the formation of an infinite sequence of exceptional points $g^{(EP)}$ such that $\lim _{g \rightarrow g^{(EP)}}\,[E_n(g)-E_{n+1}(g)]=0$.

Such a behavior of the EPs appeared to be generic. Typically, the phenomenologically relevant boundary of $\mathcal{D}_{(physical)}$ only contained, in the vast majority of the elementary closed-system models, a single isolated EP singularity. A richer, multi-parametric structure of the Hamiltonian appeared necessary for the realization of any more interesting scenario.

In order to avoid the loss of the easy mathematical tractability of the desirable toy models we decided to redirect our attention from the differential Hamiltonians $H = -\triangle + V(x)$ to their difference-operator analogues. The most straightforward implementation of such an idea is easy: One simply replaces the continuous real line of coordinates $x \in {\mathbb {R}}$ by an equidistant lattice of grid points

9 $$\begin{aligned} x_{k+1}=x_{k}+h\,, \ \ \ \ \ \ \ \ k = 0,1, \ldots , N \,. \end{aligned}$$

This opens the possibility of replacement of the conventional differential Schrödinger equation by its difference-equation analogue

10 $$\begin{aligned} - \frac{\psi (x_{k+1})-2\,\psi (x_{k})+\psi (x_{k-1})}{h^2} +V(x_k) \,\psi (x_{k}) =E\,\psi (x_k)\,, \ \ \ \ k = 1,2, \ldots , N \,. \end{aligned}$$

With the equally conventional Dirichlet asymptotic boundary conditions $\psi (x_0)=\psi (x_{N+1})=0$ the construction of bound states is then reduced to the mere linear algebraic problem

11 $$\begin{aligned} \left( \begin{array}{ccccc} v_1&{}-1&{}&{}&{}\\ -1&{}v_2&{}-1&{}&{}\\ &{}-1&{}\ddots &{}\ddots &{}\\ &{}&{}\ddots &{}v_{N-1}&{}-1\\ &{}&{}&{}-1&{}v_N \end{array} \right) \, \left( \begin{array}{c} \psi _1\\ \psi _2\\ \vdots \\ \psi _N \end{array} \right) =F\, \left( \begin{array}{c} \psi _1\\ \psi _2\\ \vdots \\ \psi _N \end{array} \right) \,. \end{aligned}$$

In this local-interaction model the Hamiltonian contains just an $N$-plet of the dynamics-determining diagonal matrix elements $v_k=h^2V(x_{k})$ yielding the spectrum of the re-scaled and shifted bound-state energies $$F_n=h^2E_n-2\ $$ with $n=0,1,\ldots , N-1$.

Our interest in Eq. (11) was initially inspired by the popularity of the non-Hermitian Hamiltonians with real spectra^21,27^. Various non-analytic square-well realizations of the potentials have been studied in this direction of research^55–58^. In these analyses an important role was played by the discrete models as sampled by Eq. (11)^59–62^.

In an introductory methodical remark let us pick up $N=6$ and let us consider Eq. (11) with one of the most elementary constant-interaction Hamiltonians

$$\begin{aligned} H^{(6)}(w)= \left[ \begin{array}{cccccc} -iw&{}-1&{}0&{}0&{}0&{}0 \\ -1&{}-iw&{}-1&{}0&{}0&{}0\\ 0&{}-1&{}-iw&{}-1&{}0&{}0 \\ 0&{}0&{}- 1&{}iw&{}-1&{}0\\ 0&{}0&{}0&{}-1&{}iw&{}-1\\ 0&{}0&{}0&{}0 &{}-1&{}iw\end{array} \right] \,. \end{aligned}$$

The brute-force numerical analysis reveals that in spite of the non-Hermiticity of the matrix, its spectrum is real (i.e., in principle, observable) inside a unique unitarity-compatible interval of

$$\begin{aligned} w \in \mathcal{D}_{(physical)} \approx (-0.322,0.322). \end{aligned}$$

Along the whole real line of parameters the model supports the existence of as many as four separate exceptional points, viz.,

$$\begin{aligned} \{- 0.54006,\,-0.32215,\,0.32215,\,0.54006\}\,. \end{aligned}$$

The distance of the outer pair of these EPs from $\mathcal{D}_{(physical)}$ is not zero so that they cannot play any immediate physical role. Their existence is only considered interesting in mathematics (or in the open-system physical setup) where people are trying to describe, irrespectively of the condition of unitarity, the whole spectrum.

## Realization: models with two free parameters

Most of the above-mentioned studies confirmed the expectations that at the sufficiently small non-Hermiticities the spectra of the energy eigenvalues should be real^21,22,63^. In our present paper we intend to complement these results by the study of models in which, via the manipulation of the EPs, one could control the qualitative dynamics directly. In a search for such models one intends to control the positions of EPs using several independent variable parameters. In such a project the main obstacles would be technical because besides a few most elementary matrix structures the brute-force numerical localization of the EPs is a badly ill-conditioned problem^64–66^.

This being said, the comparatively transparent and feasible study of the EPs can still be based on Schrödinger Eq. (11) in which almost all of the matrix elements $v_k=h^2V(x_{k})$ of the local interaction term would be assumed to vanish. In our present paper we will study, first of all, the two-parametric family of Hamiltonians

12 $$\begin{aligned} H^{(N)}(\varrho ,w)= \left[ \begin{array}{ccccc|ccccc} -\mathrm{i}{{} \varrho }&{}-1&{}0&{}\ldots &{}0&{}0&{}\ldots &{} &{}\ldots &{}0 \\ -1&{}{{} {0}}&{}-1&{}\ddots &{}\vdots &{}\vdots &{}\ddots &{}&{}&{}\vdots \\ 0 &{}\ddots &{}\ddots &{}\ddots &{}0&{}\vdots &{}&{}&{}&{} \\ \vdots &{}\ddots &{}-1&{}{{} {0}}&{}-1&{}0&{}&{}&{}\ddots &{}\vdots \\ 0&{}\ldots &{}0&{}-1&{}- \mathrm{i}\,w&{}-1&{}0&{}\ldots &{}\ldots &{}0 \\ \hline 0&{}\ldots &{}\ldots &{}0&{}-1&{}\mathrm{i}\,w&{}- 1&{}0&{}\ldots &{}0 \\ \vdots &{}\ddots &{}&{}&{}0&{}-1&{}{{} {0}}&{}-1&{}\ddots &{}\vdots \\ &{}&{}&{}&{}\vdots &{}0&{}\ddots &{}\ddots &{}\ddots &{}0 \\ \vdots &{}&{}&{}\ddots &{}\vdots &{}\vdots &{}\ddots &{}-1&{}{{} {0}}&{}-1 \\ {}0&{}\ldots &{}&{}\ldots &{}0&{}0&{}\ldots &{}0&{}-1&{} \mathrm{i}{{} \varrho } \end{array} \right] \,. \end{aligned}$$

in which $N=2K$ is even and in which the central and remote parts of the interaction (with the respective strengths *w* and $\varrho$) are well separated.

### The confluence of EPs controlled by the fine-tuning of the remote gain-and-loss interaction

Technically, the separation of the influence of *w* and $\varrho$ can simply be strengthened, whenever needed, by the choice of a sufficiently large matrix dimension *N*. At the same time, the potentially adverse aspect of the growth of *N* (making the secular equation less easily tractable) can very easily be suppressed using the dedicated $N$-independent matching method of Ref.^62^. Using this method one can always try to test whether the bound-state spectrum of the closed-system toy-model Hamiltonian (12) is real.

Usually, the answer becomes affirmative for the parameters lying inside a two-dimensional unitarity-compatible (and, say, real) domain $\mathcal{D}_{(physical)}$. Within the framework of our present project we will only be interested in the situations in which one of the parameters is fixed while the other one approaches the boundary $\partial \mathcal{D}_{(physical)}$ of the energy-reality domain. What one then *a priori * expects is that for the different choices of the fixed parameter the mergers of the energies encountered at the EP boundary might be of different types.

**Figure 2:**
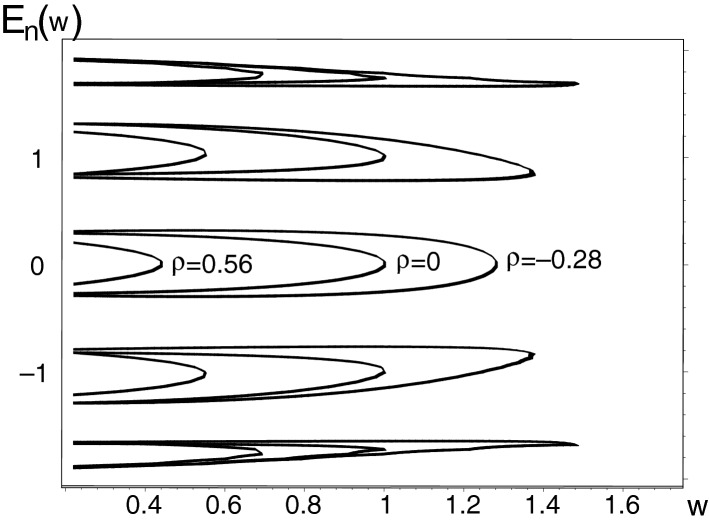
The $N$-plets of the real energy eigenvalues $E_n({\varrho },w)$ of Hamiltonian (12) with $N=10$ and $n=0,1,\ldots ,N-1$ at constant ${\varrho }=-0.28$ (the rightmost curves), at constant ${\varrho }=0$ (the curves in the middle) and at constant ${\varrho }=0.56$ (the leftmost curves). Picture created using Maple^44,45^.

In the first test of the hypothesis let us choose $N=10$. Once we fix the remote-gain-and-loss parameter ${\varrho }$ we may study the spectra of energies $E_n({\varrho },w)$, $n=0,1,\ldots ,N-1$ as functions of the remaining variable parameter *w*. Numerically we evaluated several characteristic samples of such a type. In Fig. 2 we displayed three of them. The energies are shown there as functions of *w*, calculated at the three different values of the remote-non-Hermiticity parameter ${\varrho }$, viz., at ${\varrho }=-0.28$ (the rightmost curves), of ${\varrho }=0$ (the middle-positioned curves), and of ${\varrho }=0.56$ (the leftmost curves). After inspection of this picture it is possible to formulate the following observation.

#### **Conjecture 1**

*At the sufficiently small real values of the remote-interaction parameter* ${\varrho }$ *the separate EP2 coordinates* $w=w^{(EP2)}_j({\varrho })$ *of the pairwise mergers of the neighboring energies* $E_{2j}^{(10)}({\varrho },w)$ *and* $E_{2j+1}^{(10)}({\varrho },w)$ *are all strictly decreasing functions of* ${\varrho }$ *such that*

13 $$\begin{aligned} w^{(EP2)}_2({\varrho })\le w^{(EP2)}_1({\varrho })= w^{(EP2)}_3({\varrho })\le w^{(EP2)}_0({\varrho })= w^{(EP2)}_4({\varrho })\,. \end{aligned}$$

*The inequalities become sharp at* ${\varrho } \ne 0$*. At larger* $w>w^{(EP2)}_j({\varrho })$ *the respective pairs of energies become complex so that the local boundary of* $\mathcal{D}_{(physical)}$ *becomes determined by function* $w^{(EP2)}_2({\varrho })$.

Beyond such a purely numerically supported hypothesis (which could probably be generalized to hold at any matrix dimension $N=2K$), the inspection of Fig. 2 also inspired the formulation of the following exact result valid at all *K*s.

#### **Proposition 2**

*During the passage of the remote coupling* $\,\varrho \,$ *through the origin at* $\varrho =0$*, Hamiltonian (*12*) encounters the instantaneous confluence of all of the separate exceptional points of order two,*

14 $$\begin{aligned} w^{(EP2)}_j(0)=1\,, \ \ \ j = 0,1,\ldots ,N-1\,. \end{aligned}$$

*The canonical form of the* $w \rightarrow 1$ *limit of matrix* (12) *then acquires the* *N* *by* *N* *matrix form*

15 $$\begin{aligned} H^{(2K)}(0,1)\ \sim \ \bigoplus _{j=1}^K\, J^{(2)}(\eta _j) \end{aligned}$$

*of a direct sum of* *K* *two-dimensional Jordan blocks as defined in Eq.* (7).

In the limit of ${\varrho } \rightarrow 0$, we witness the complete degeneracy *alias * confluence of all of the separate EP2s. The rigorous proof will be given in section 5 below. As a byproduct of this proof, also the values of the limiting energies $\eta _j$ will be shown obtainable in closed form.

### The confluence of EPs caused by the fine-tuning of the central gain-and-loss interaction

Our above-outlined projection of the motion of the EP boundaries of the two-dimensional domain $\mathcal{D}_{(physical)}$ can be complemented by the perpendicular sections in which the value of *w* is fixed. We may expect that at the boundary $\partial \mathcal{D}_{(physical)}$ the energies will merge in a way reflecting the characteristics of the underlying EPs.

**Figure 3:**
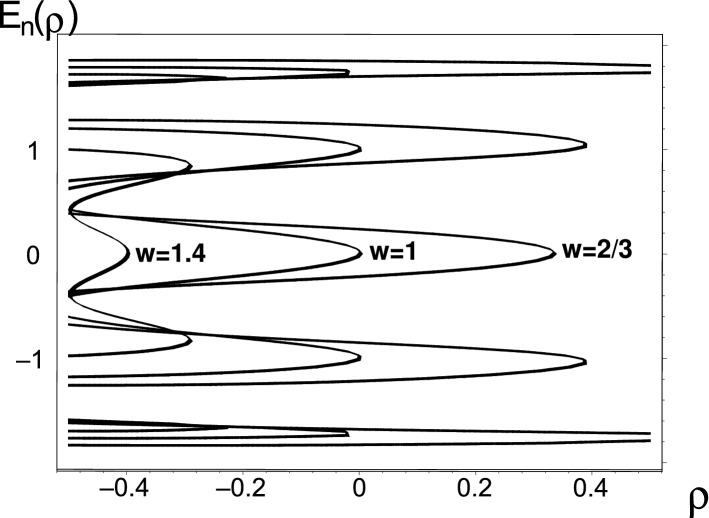
The $$N$$-plets of the real energy eigenvalues $$E_n({\varrho })$$ of Hamiltonian (12) with $$N=10$$ and $$n=0,1,\ldots ,N-1$$ at constant $${w}=2/3 $$ (the rightmost curves), at constant $${w}=1 $$ (the curves in the middle) and at constant $${w}=1.4 $$ (the leftmost curves). Picture created using Maple^44,45^.

The resulting scenario sampled in Fig. 3 is qualitatively not too different from its predecessor of Fig. 2. At every fixed value of *w*, the single central EP2 energy-merger ${\varrho }^{(EP)}_2$ is smaller than its first off-central doubly-degenerate partner ${\varrho }^{(EP)}_1={\varrho }^{(EP)}_3$ which is, in its turn, smaller than the second, most off-central partner doublet ${\varrho }^{(EP)}_0={\varrho }^{(EP)}_4$. With the growth of *w* sampled, in the picture, by the choice of $w=2/3$, $w=1$ and $w=1.4$, all of the energy mergers move leftwards.

#### **Conjecture 3**

*In a vicinity of* $w=1$ *the separate EP2 coordinates *$\varrho =\varrho ^{(EP2)}_j({w})$ *of the pairwise mergers of the neighboring energies* $E_{2j}^{(10)}({\varrho },w)$ *and* $E_{2j+1}^{(10)}({\varrho },w)$ *are all strictly decreasing functions of* *w* *such that*

16 $$\begin{aligned} \varrho ^{(EP2)}_2({w})\le \varrho ^{(EP2)}_1({w})= \varrho ^{(EP2)}_3({w})\le \varrho ^{(EP2)}_0({w})= \varrho ^{(EP2)}_4({w})\,. \end{aligned}$$

*The inequalities are certainly sharp a*t ${w} \ne 1$*. At the small and positive difference* $\varrho -\varrho ^{(EP2)}_j({w})>0$ *the respective pairs of energies cease to be real so that the local boundary of* $\mathcal{D}_{(physical)}$ *is prescribed by function* $\varrho ^{(EP2)}_2({w})$.

In the limit of $w \rightarrow 1$ all of the EP2 singularities may be guessed to coincide forming a degenerate quintuplet EP10. Such a possibility advised by the inspection of Fig. 3 inspired the following proposition in which the value of the even matrix dimension $N=2K$ can be arbitrary.

#### **Proposition 4**

*During the passage of the central coupling* $\,w\,$ *through the value of* $w=w^{(EP)}=1$*, Hamiltonian* (12) *encounters the instantaneous confluence of all of the separate exceptional points of order two,*

17 $$\begin{aligned} \varrho ^{(EP2)}_j(1)=0\,, \ \ \ j = 0,1,\ldots ,N-1\,. \end{aligned}$$

*The canonical form of the* $\varrho \rightarrow 0$* limit of matrix* (12) *acquires the same* *N* *by* *N* *matrix form* (15) *as in Proposition* 2.

#### *Proof*

In the light of Proposition 2 the proof is elementary because our Hamiltonian matrix $H^{(2K)}(\varrho ,w)$ can be transformed into matrix $H^{(2K)}(w,\varrho )$ using an elementary block-diagonal unitary-transformation matrix $\mathcal{U}^{(2K)}$ defined as a direct sum of two *K* by *K* antidiagonal unit matrices $\mathcal{I}^{(K)}$ with Kronecker-delta elements $\mathcal{I}^{(K)}_{i,j} ={\delta }_{i,K+1-i}$, $i=1,2,\ldots ,K$. $\square$

## Exact solutions at $\varrho =0$

Our illustrative Hamiltonian (12) is the special case of a broader class of models of Eq. (11). Some of them might be analytically solvable by the matching method of Ref.^62^. What would be required is a special choice of the matrix elements $v_j$ of the interaction. For the sake of simplicity we decided to consider just the very special model (12), with the study of its possible generalizations left to the readers.

### Constructive proof of Proposition 2

The independent variability of the two real parameters $\varrho$ and *w* in (12) proved sufficient for our present illustration purposes. Now we only have to prove Proposition 2 in which our $\varrho =0$ toy-model Hamiltonian has even $N=2J+2$ and mere two nonzero values of $v_j$,

18 $$\begin{aligned} H^{(2J+2)}(w)= \left( \begin{array}{cccc|cc|cccc} 0&{}-1&{}0&{}\ldots &{}0&{}0&{}0&{}\ldots &{}\ldots &{}0 \\ {} -1&{}0&{}-1&{}\ddots &{}\vdots &{}\vdots &{}\vdots &{}&{}&{}\vdots \\ {} 0&{}-1&{}\ddots &{}\ddots &{}0&{}\vdots &{}&{}&{}&{} \\ {} \vdots &{}\ddots &{}\ddots &{}0&{}-1&{}0&{}\vdots &{}&{}&{}\vdots \\ \hline {}0&{}\ldots &{}0&{}-1&{}-\mathrm{i}w&{}-1&{}0&{}\ldots &{}\ldots &{}0 \\ {}0 &{}\ldots &{}\ldots &{}0&{}-1&{}\mathrm{i}w&{}-1&{}0&{}\ldots &{}0 \\ \hline {}\vdots &{}&{}&{}\vdots &{}0&{}-1&{}0&{}-1&{}\ddots &{}\vdots \\ {} &{}&{}&{}&{}\vdots &{}0&{}-1&{}\ddots &{}\ddots &{}0 \\ {} \vdots &{}&{}&{}\vdots &{}\vdots &{}\vdots &{}\ddots &{}\ddots &{}0&{}-1 \\ {} 0&{}\ldots &{}\ldots &{}0&{} 0&{}0&{}\ldots &{}0&{}-1&{}0 \end{array} \right) \end{aligned}$$

The related Schrödinger equation is tractable by the standard numerical diagonalization techniques. The task becomes simplified when one introduces, in the spirit of Refs.^19,67^, the requirement $\mathcal{PT}\,H^{(2J+2)}(w)=H^{(2J+2)}(w)\,\mathcal{PT}$ of $\mathcal{PT}$-symmetry defined in terms of the antidiagonal-unit matrix $\mathcal{P}$ [changing the parity and causing the left-right inversion of the spatial lattice (9)] and of the antilinear complex-conjugation operator $\mathcal{T}$ (which simulates the time reversal in Schrödinger equation). Whenever our real parameter *w* is such that the spectrum remains real and non-degenerate, Schrödinger equation (11) will then yield, exclusively, just the $\mathcal{PT}$-symmetric eigenstates,

19 $$\begin{aligned} \mathcal{PT}\, \left( \begin{array}{c} \psi _1\\ \psi _2\\ \vdots \\ \psi _N \end{array} \right) \sim \left( \begin{array}{c} \psi _1\\ \psi _2\\ \vdots \\ \psi _N \end{array} \right) \,. \end{aligned}$$

We may set $\psi _N=\psi _1^*$, $\psi _{N-1}=\psi _2^*$ (etc), we may abbreviate $h^2E=-2x=-2\,\cos \theta$, and we may recall the definition of the Tshebyshev plynomials of the second kind,

20 $$\begin{aligned} U_k(\cos \theta )=\frac{\sin (k+1)\theta }{\sin \theta }\,, \ \ \ \ k = 0, 1, \ldots \,. \end{aligned}$$

The recurrences satisfied by these polynomials^68,69^ enable us to guess the ansatz

$$\begin{aligned} \psi _{k+1}=(\alpha +\mathrm{i}\,\beta )\,U_k(x)\,, \ \ \ \ k = 0, 1, \ldots , J\, \end{aligned}$$

containing just a pair of unspecified real parameters $\alpha$ and $\beta$. Its use converts the first *J* lines of relations (11) into identities. Recalling the $\mathcal{PT}$-symmetry of the model we may also write down the rest of the components of the eigenvector in closed form reflecting the validity of the last *J* lines of relations (11),

$$\begin{aligned} \psi _{N-k}=(\alpha -\mathrm{i}\,\beta )\,U_k(x)\,, \ \ \ \ k = 0, 1, \ldots , J\,. \end{aligned}$$

What remains to be satisfied are the two middle lines of Schrödinger equation (11),

$$\begin{aligned}&-(\alpha +\mathrm{i}\,\beta )\,U_{J-1}(x) +[(2x-\mathrm{i}w)\,(\alpha +\mathrm{i}\,\beta )-(\alpha -\mathrm{i}\,\beta )]\, U_{J}(x)=0\,, \\&\quad -(\alpha -\mathrm{i}\,\beta )\,U_{J-1}(x) +[(2x+\mathrm{i}w)\,(\alpha -\mathrm{i}\,\beta )-(\alpha +\mathrm{i}\,\beta )]\, U_{J}(x)=0\,. \end{aligned}$$

The separation of the real and imaginary components yields

$$\begin{aligned} -\alpha \,U_{J-1}(x) +(2x\alpha +w\,\beta )-\alpha )\, U_{J}(x)=0\, \end{aligned}$$

and

$$\begin{aligned} -\beta \,U_{J-1}(x) +(2x\beta -w\,\alpha +\beta )\, U_{J}(x)=0\,. \end{aligned}$$

After a premultiplication by suitable constants, the sum of the latter two relations yields

21 $$\begin{aligned} -2\,\alpha \,\beta \,U_{J-1}(x)+[4\,\alpha \,\beta \,x +(\beta ^2-\alpha ^2)\,w]\,U_{J}(x)=0 \end{aligned}$$

while their difference only leads to elementary relation

22 $$\begin{aligned} (\alpha ^2+\beta ^2)\,w=2\,\alpha \,\beta \,. \end{aligned}$$

This enables us to reparametrize $\alpha =\alpha (\tau )=\cos \tau$ and $\beta =\beta (\tau )=\sin \tau$ and to deduce that $w=w(\tau )=\sin 2\tau$.

One can treat the auxiliary angle $\tau$ as an alternative dynamical-input information about the strength of the non-Hermiticity. We are now only left with the secular equation (21), i.e.,

23 $$\begin{aligned} 2\,\alpha \,\beta \,U_{J+1}(x) +(\beta ^2-\alpha ^2)\,w\,\,U_{J}(x)=0\,. \end{aligned}$$

The insertion of *w* reduces it to the relation

24 $$\begin{aligned} U_{J+1}(x)= [\alpha ^2(\tau )-\beta ^2(\tau )]\,U_{J}(x)\,. \end{aligned}$$

This is our ultimate implicit definition of the spectrum of the energies $h^2E=-2x$ at arbitrary matrix dimension $N=2J+2$.

At the $\mathcal{PT}$-symmetry-breakdown boundaries with $w=w^{(EP)}=\pm 1$ or $\tau =\tau ^{(EP)}=\pm \pi /4$, we have $\alpha ^2(\tau ^{(EP)})=\beta ^2(\tau ^{(EP)})$ so that the EPN-related energy values coincide with the roots of a single polynomial,

25 $$\begin{aligned} U_{J+1}\left( x^{(EP)}\right) =0\,. \end{aligned}$$

These roots can be given an elementary form given by formula (20).

**Figure 4:**
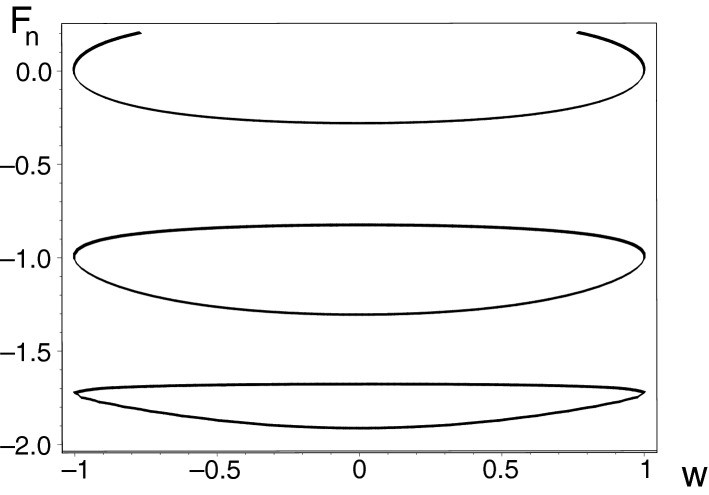
The left-right symmetry of the low-lying spectrum of model (18) at $N=10$. The spectral locus is also symmetric with respect to the $F=0$ axis, so we did not need to display the upper, high-excitation half of the spectrum. Picture created using Maple^44,45^.

The availability of such an explicit parameter-dependence of the spectrum in the EPN limit can be extended to cover also, in an approximative form, a small vicinity of the singularity. In this vicinity the difference $\alpha ^2(\tau )-\beta ^2(\tau )$ entering Eq. (24) will be a small number. The well known intertwining property of the roots of the polynomials $U_{J+1}(x)$ and $U_{J}(x)$ will then immediately imply the correct qualitative understanding of the branching of the levels at $|w| \lessapprox 1$ as sampled, at $N=10$, in Fig. 4.

### Spectral curves at $N=10$

The interval $w \in (-1,1)$ of the unitarity-compatible physical parameters is the same for *all * of the bound states at an *arbitrary * even matrix dimension $N=2J+2$. Whenever the value of *w* leaves this interval, *all * of the energies cease to be real so that abruptly, the *whole * spectrum becomes unobservable. At all of the lattice-size-determining integers *J*, even the parameter-dependence of the numerically evaluated spectra remains qualitatively the same, characterized by the specific *pairwise * degeneracies of *all * of the energy levels at $w=1$ and $w=-1$.

At $N=10$ the model still remains non-numerical. Its secular polynomial $P^{(10)}({F},{w})$ is a polynomial of the fifth degree in the energy-representing variable $F^2$,

26 $$\begin{aligned} {{ {F}}}^{10}+ \left( -9+{{ {w}}}^{2} \right) {{ {F}}}^{8}+ \left( 28-6\,{{ {w}}}^{2} \right) {{ {F}}}^{6}+ \left( -35+11\,{ { {w}}}^{2} \right) {{ {F}}}^{4}+ \left( 15-6\,{{ {w}}}^{2} \right) {{ {F}}}^{2}-1+{{ {w}}}^{2}\, \end{aligned}$$

but this does not imply that the search for its roots is complicated. Their brute-force numerical localization is not necessary. It is sufficient to notice that the secular polynomial is just a linear function of the square of the coupling constant $w^2$. This implies that the spectrum can be constructed, in the implicit-function form, non-numerically.

The shape and symmetry of the spectrum are sampled in Fig. 4. The picture just reconfirms the existence of the strictly two Kato’s exceptional points $w =w^{(EP)}_\pm = \pm 1$. One can visualize the coupling *w* as an *elementary * function

$$\begin{aligned} w=w_\pm (F) = \pm \, [\left( {{F}}^{2}-1 \right) ^2 - {{F}}^{2} ]^{-1}\, {\sqrt{1-{{F}}^{10}+9\,{{F}}^{8}-28\,{{F}}^{6}+35\,{{F}}^{4}-15\,{{F}}^{ 2}}}\, \end{aligned}$$

of the energy. At both of the EPN extremes with $|w|=1$ the $N=10$ secular polynomial can be factorized,

27 $$\begin{aligned} P^{(10)}({F},\pm 1)={F}^2\,({F}^2-1)^2\,({F}^2-3)^2\,. \end{aligned}$$

The function $w=w(F)$ can be also Taylor-expanded. Near $F=0$ this yields the symmetric and “deeper-than-quadratic” well,

$$\begin{aligned} w({F}) \approx -1+{\frac{9}{2}}{{F}}^{2} +{\frac{201}{8}}{{F}}^{4}+O \left( {{F}}^{5} \right) \,. \end{aligned}$$

Similarly, off the origin we get, in agreement with the picture, the two narrower and asymmetric wells which are steeper than the one near the origin. Thus, we get

$$\begin{aligned} w({F}) \approx -1+8\, \left( {F}-1 \right) ^{2}-24\, \left( {F}-1 \right) ^{3}+122\, \left( {F}-1 \right) ^{4}+O \left( \left( {F}-1 \right) ^{5} \right) \,, \end{aligned}$$

etc. Finally, the outer wells have just the more pronounced shapes of the same form, with

$$\begin{aligned} w({F}) \approx -1 + 72\, \left( {F}- \sqrt{3} \right) ^{2}- 648\,\sqrt{3}\, \left( {F}- \sqrt{3} \right) ^{3}+ 16782\, \left( {F}- \sqrt{3} \right) ^{4}+O \left( \left( {F}- \sqrt{3} \right) ^{5} \right) \, \end{aligned}$$

etc. All of these observations gave birth to their generalizations valid in any analogous EP-supporting $N$-level quantum system with arbitrary finite $N<\infty$.

## Discussion

From the purely methodical point of view the choice of the discrete local-interaction model of Eq. (11) has its weaknesses. Firstly, its variable parameters only lie on the main diagonal. This lowers the flexibility of dynamics leading, typically, just to the EP2 energy mergers. Secondly, additional antilinear symmetries [sampled here by $\mathcal{PT}$-symmetry of Eq. (19)] had to be imposed in order to guarantee the reality of the spectrum. Thirdly, the well known numerically ill-conditioned nature of the study of the limiting transition $g \rightarrow g^{(EP)}$ often forces us to use certain truly sophisticated construction methods in a way sampled, say, in Ref.^70^.

For all of these reasons it will make sense to turn attention to the more general matrix models in which the practical calculations remain feasible but in which it should still be possible to enhance the flexibility of the picture of the EP-related dynamics. Let us now mention a few hints for the future projects oriented in this direction.

### Parallels between harmonic oscillator and our $N<\infty$ models

The turn of attention to the more general classes of models might open new ways towards an immediate further development of the theory itself. In order to be more specific let us recall, once more, the harmonic oscillator results as sampled in Fig. 1 and in Eq. (3) above. In place of the canonical Jordan-block limit of Eq. (7)  one obtains, for them, an alternative, infinite-dimensional but partitioned Jordan-block limit

28 $$\begin{aligned} \lim _{g \rightarrow g^{(EP)}}\,H^{(HO)}(g) \sim \left( \begin{array}{cc|cc|cc} 2&{}1&{}0&{}0&{}0&{}\ldots \\ 0&{}2&{}0&{}0&{}0&{}\ldots \\ \hline 0&{}0&{}6&{}1&{}0&{}\ldots \\ 0&{}0&{}0&{}6&{}0&{}\ldots \\ \hline 0&{}0&{}0&{}0&{}10&{}\ldots \\ \vdots &{}\vdots &{}\vdots &{}\vdots &{}\ddots &{}\ddots \end{array} \right) \, \end{aligned}$$

of the form of Eq. (15) with infinite sequence of the energy mergers available in closed form, $\eta _j=4j-2$^46^.

The EP singularities of our present $N < \infty$ models (12) lead to an analogous canonical-representation limit with the known values of $\eta _j$. In particular, for our $N=10$ model (18) characterized by the secular polynomial of Eq. (26) and by the canonical form (15) of the EP10 limit with $K=5$, it would be easy to construct the so called transition matrices $Q^{{(10)}}_{}$ and to evaluate the canonical-representation form of the Hamiltonian,

29 $$\begin{aligned} H^{{(canonical)}}_{}(w)\, =[Q^{{(10)}}_{}]^{-1} \,H^{{(10)}}(w)\, \cdot Q^{{(10)}}_{} \, \end{aligned}$$

In the EP limit we would get

30 $$\begin{aligned} \lim _{w \rightarrow w^{(EP)}}\,H^{(10)}(w) \sim H^{{(canonical)}}_{}(1)= \left( \begin{array}{cc|cc|cc|cc|cc} -\sqrt{3}&{}1&{}&{}&{}&{}&{}&{}&{}&{}\\ 0&{}-\sqrt{3}&{}&{}&{}&{}&{}&{}&{}&{}\\ \hline &{}&{}-1&{}1&{}&{}&{}&{}&{}&{}\\ &{}&{}0&{}-1&{}&{}&{}&{}&{}&{}\\ \hline &{}&{}&{}&{}0&{}1&{}&{}&{}&{}\\ &{}&{}&{}&{}0&{}0&{}&{}&{}&{}\\ \hline &{}&{}&{}&{}&{}&{}1&{}1&{}&{}\\ &{}&{}&{}&{}&{}&{}0&{}1&{}&{}\\ \hline &{}&{}&{}&{}&{}&{}&{}&{}\sqrt{3}&{}1\\ &{}&{}&{}&{}&{}&{}&{}&{}0&{}\sqrt{3}\\ \end{array} \right) \,. \end{aligned}$$

Such a canonical-Hamiltonian matrix is block-diagonal. At a fixed algebraic multiplicity of EPN (i.e., at $N=10$ in this case) the number *K* of its independent eigenvectors (called the geometric multiplicity of EPN) is maximal (here, we have $K=5$).

### Asymmetric real-matrix models

One of the next-step model-building strategies could be inspired by the less explored non-numerical constructions of Refs.^71,72^. The necessary simplification of the technicalities has been achieved there by the reduction of the class of the eligible Hamiltonians to the mere tridiagonal real and real-parameter-dependent asymmetric matrices admitting off-diagonal interaction terms. In contrast to our preceding models, the weakly non-local interactions of such a type proved useful, e.g., in the pseudo-Hermitian models of scattering^73–78^.

For an illustration of their specific merits let us recall now the six-by-six-dimensional special case of the *N* by *N* matrices of Ref.^71^, and let us complement it by an $\mathcal{O}(g)$ perturbation. This leads to one of the most user-friendly real-matrix two-parametric Hamiltonians, viz.,

31 $$\begin{aligned} H^{(toy)}(g,\lambda )=\left[ \begin{array}{cccccc} -5+g &{}\sqrt{5+5\,{ \lambda }}&{}0&{}0&{}0&{}0 \\ -\sqrt{5+5\,{ \lambda }}&{}-3&{}2\,\sqrt{2+2\,{ \lambda }}&{}0&{}0&{}0 \\ 0&{}-2\,\sqrt{2+2\,{ \lambda }}&{}-1&{}3\,\sqrt{ 1+{ \lambda }}&{}0&{}0 \\ 0&{}0&{}-3\,\sqrt{1+{ \lambda }}&{}1&{}2\, \sqrt{2+2\,{ \lambda }}&{}0 \\ 0&{}0&{}0&{}-2\,\sqrt{2+2\,{ \lambda }}&{}3&{}\sqrt{5+5\,{ \lambda }} \\ 0&{}0&{}0&{}0&{}-\sqrt{ 5+5\,{ \lambda }}&{}5-g\end{array} \right] \,. \end{aligned}$$

The real, non-degenerate and equidistant spectrum is obtained in a $g$-dependent unitarity-compatible interval $\mathcal{D}^{(toy)}(g)$ of parameters $\lambda$. The simplest proof becomes available in the unperturbed case with $g=0$. One merely has to recall the closed formulae of Ref.^71^ yielding the admissibility interval of $\lambda \in (-\infty ,0)$ or, in the real-matrix case, the narrower range of $\lambda \in (-1,0)$.

### The parameter-controlled change of the geometric multiplicity

**Figure 5:**
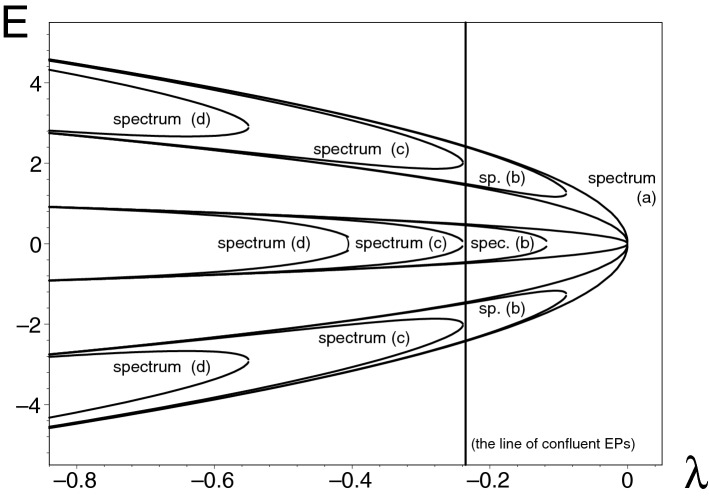
The $\lambda$-dependent spectra of Hamiltonian (31) at $g=0$ [spectrum (a)], $g=1/500$ [spectrum (b)], $g=1/40$ [spectrum (c)] and $g=1/5$ [spectrum (d)]. Picture created using Maple^44,45^.

In model (31) with small $g>0$ we may omit, as trivial, the half-line of parameters $\lambda < -1$ at which the matrix becomes complex but Hermitian. We are left with the variability of the single unitarity-supporting parameter $\lambda \in (-1,\mu (g))=\mathcal{D}^{(toy)}(g)$ with $\mu (g) \le 0$, and we notice that the parameter-dependence of the spectrum is entirely different from our preceding models. At the left boundary $\lambda =-1$ the matrix $H^{(toy)}(g,-1)$ becomes diagonal, i.e., Hermitian and tractable as a truncated conventional harmonic oscillator with equidistant spectrum. In contrast, the spectral pattern is very different at the right boundary of $\mathcal{D}^{(toy)}$: see Fig. 5 for illustration.

The comparatively elementary nature of the model facilitates a detailed interpretation of the shapes of the spectra. At the smallest *g*s the value of the upper bound $\mu (g)$ is determined by the central energy merger representing an EP of order two (EP2). We may set $\mu (g)= \lambda ^{(EP)}_1$. In the opposite extreme of a large shift *g* one gets another, different behavior and formula for $\mu (g)= \lambda ^{(EP)}_0= \lambda ^{(EP)}_2$. The change of the pattern clearly reflects the fragility of the off-cental states exhibiting, at larger *g*s, the confluence of their EP2 singularities (in the notation of Ref.^79^ one would write EP=EP2$\oplus$EP2).

The main *qualitative * difference from the dynamics of the “local” models (12) can be now formulated as the following observation.

#### **Conjecture 5**

*In model* (31) *the separate generic* $g$-*dependent EP2 coordinates* $\lambda =\lambda ^{(EP2)}_j({g})$ *of the pairwise mergers of the neighboring energies* $E_{2j}^{(10)}({\lambda },g)$ *and* $E_{2j+1}^{(10)}({\varrho },g)$ *with* $j=0, 1,2$ *are all strictly decreasing functions of* ${g} \in (0,1/5)$ *such that*

32 $$\begin{aligned} \lambda ^{(EP2)}_1({g})< \lambda ^{(EP2)}_0({g})= \lambda ^{(EP2)}_2({g})<0\, \end{aligned}$$

*near the origin (i.e., for* $g\ll 1/40$*), and such that*

33 $$\begin{aligned} 0> \lambda ^{(EP2)}_1({g})> \lambda ^{(EP2)}_0({g})= \lambda ^{(EP2)}_2({g})\, \end{aligned}$$

*at the larger* $g \gg 1/40$.

From this bracketing feature one can immediately deduce the following obvious result which we will give here without a formal proof.

#### **Proposition 6**

*In model* (31)* there exists an EP6 = EP2* $\oplus$ *EP2* $\oplus$ *EP2 singularity* $g^{(EP6)} \in (0,1/5)$ *with the geometric multiplicity* $K=3$.

The approximate numerical estimate of $g^{(EP6)} \approx 1/40$ has been used to display the corresponding $\lambda$-dependence of the energies in Fig. 5 [marked as “spectrum (c)”]. Obviously, the local boundary of the physical interval $\mathcal{D}^{(toy)}$ is given by the function $\lambda ^{(EP2)}_1({g})$ at $g<g^{(EP6)}$, and by the function $\lambda ^{(EP2)}_0({g})=\lambda ^{(EP2)}_2({g})$ at $g>g^{(EP6)}$.

We may conclude that our toy model (31) admits a smooth transition from the dynamical regime with the minimal geometric multiplicity $K=1$ of the EP6 at $g=g^{(EP6)}_{(K=1)} =0$ to its maximal-geometric-multiplicity alternative with $K=3$ at $g=g^{(EP6)}_{(K=3)} \approx 1/40$. In the former case all of the bound-state energies converge, in a way prescribed by Eq. (6), to the single EP value $\eta =E^{(EPN)}$ with $N=6$ at $\lambda ^{(EP)}=\lambda ^{(EP)}(g)=\lambda ^{(EP)}(0)$,

34 $$\begin{aligned} \lim _{{\lambda }\rightarrow {\lambda }^{(EP)}} E_{j}({\lambda })=E^{(EP6)}\,,\ \ \ \ j=0,1,\ldots ,5\,. \end{aligned}$$

In^71^, via solvable tridiagonal real-matrix models we managed to simulate such a minimal geometric multiplicity behavior of the energies for an arbitrary preselected finite Hilbert-space dimension $N < \infty$. Using a brute-force numerical search such a type of construction with minimal $K=1$ remains feasible even in the models which are realistic^80^.

In the opposite extreme of the dynamical scenario near EPN = EP2 $\oplus$ EP2 $\oplus \ldots \oplus$ EP2 with the even algebraic EP multiplicity $N=2K$ (such that *K* now represents the maximal geometric multiplicity) the energy degeneracy is “maximally incomplete”, having proceeded merely pairwise,

35 $$\begin{aligned} \lim _{{\lambda }\rightarrow {\lambda }^{(EP)}} E_{n_j}({\lambda })=\eta _j\,,\ \ \ \ j=1,2,\ldots ,K\,, \ \ \ \ n_1=0,1\,,\ \ n_2=2,3\,,\ \ \ldots \,\ \ n_K=2K-2,2K-1\,. \end{aligned}$$

For our model (31) we just have to insert $K=3$ and specify $\lambda ^{(EP)} =\lambda ^{(EP)}\left( g^{(EP6)}_{(K=3)}\right)$.

Along similar lines one can simulate the genuine quantum phase transition phenomena with an optional geometric multiplicity *K*. The first applications of such an approach may already be found in the elementary methodical toy models^81^, with the next stage of developments to be aimed at the topical realistic applications of the theory, say, in the descriptions of the mechanism of the Bose-Einstein condensation using the multi-bosonic pseudo-Hermitian Bose-Hubbard Hamiltonians^79,82–84^.

Multiple related mathematical questions remain open. Nevertheless, using the standard Kato’s terminology^18^, we certainly will have to distinguish, at a fixed algebraic EPN multiplicity *N*, between the occurrence of a minimal geometric multiplicity $K=1$ [leading to the canonical-representation limit of Eq. (7)], of a maximal geometric multiplicity $K=N/2$ [yielding the alternative canonical-representation limit of Eq. (15)], and of all of the other possibilities in between these two extremes. This leads us to our final methodical conclusion.

#### **Proposition 7**

*Any given EPN-supporting quantum closed-system Hamiltonian may be characterized, in its EPN limit, by its canonical* *N* *by* *N* *matrix form* $H^{{(canonical)}}_{}$ *with the most general direct-sum* *alias* *block-diagonal-matrix structure*

36 $$\begin{aligned} H^{{(canonical)}}_{}= \bigoplus _{j=1}^K\, J^{(N_j)}(\eta _j)\,, \ \ \ \ N_1+N_2+\ldots N_K=N\, \end{aligned}$$

*containing nontrivial partitions* $N_j \ge 2$.

The latter operator EPN limit is fully characterized by the partitioning of *N* (check some of its number-theory aspects in^85^) and by the $K$-plet of the EPN energies $\eta _j$. Thus, every classification of phase transitions should refer to the pair of the multiplicites *N* (algebraic) and *K* (geometric). The above-studied minimal- and maximal-$K$ models also become reclassified as the two extreme special cases which are, in some sense, just most elementary.

### Outlook

The phenomenology-oriented core of our present message is that one of the most promising innovative means of the control of unitary quantum dynamics should be sought in a purposeful manipulation with the Kato’s exceptional points $g^{(EP)}$.

At the first sight such a statement sounds like an oxymoron because the unitarity of the evolution requires, in Schrödinger picture, the self-adjointness of the Hamiltonian, while such a requirement is *manifestly * violated by *H*(*g*) at $g=g^{(EP)}$. For this reason it is necessary to emphasize the existence of the two tacit assumptions behind the PHQM SP theory. The first one is that we really *exclude * the singularity $g=g^{(EP)}$, and that we only work in its vicinity $\mathcal{D}_{(physical)}$, with the parameter *g* admitted to lie arbitrarily close to $g^{(EP)}$ (i.e., formally, $g^{(EP)} \in \partial \mathcal{D}_{(physical)}$).

The second tacit assumption is more standard and means the acceptance of the currently very popular PHQM update of quantum theory. In this framework the self-adjointness of *H*(*g*) is considered $g$-dependent or, more precisely, metric-operator-dependent, $\Theta (g)$-dependent. Precisely due to this freedom, the desirable limiting transition $g\rightarrow g^{(EP)}$ can always be performed in a mathematically consistent and unitarity-compatible manner.

In the current literature, unfortunately, the EP-related field of phenomenology is predominantly developed in its applications to the open (i.e., in other words, manifestly non-unitary) quantum systems^31^ and/or to various non-quantum or even non-linear systems^26,27,86,87^. One of the reasons is that in such a setup the model-building process is technically easier, being still allowed to work with the trivial choice of $\Theta (g)=I$.

In this sense, we tried to lower here the related psychological barriers. Having emphasized the fundamental aspect of the strictly unitary theory (requiring, typically, nontrivial metrics $\Theta (g)\ne I$), we illustrated its user-friendly nature by a detailed analysis of certain finite-dimensional *N* by *N* matrix benchmark Hamiltonians $H^{(N)}(g)$.

The validity of our conclusions remains model-independent of course. In their brief summary let us emphasize that one of the key benefits of the PHQM formulation of the theory lies in its capability of covering multiple apparently exotic system-evolution scenarios in which *g* is not too far from $g^{(EP)}$. Then, the metric $\Theta (g)$ becomes very different from the conventional choice of $\Theta (g)=I$ of textbooks. Besides an undeniable phenomenological appeal of the anisotropy $\Theta (g)\ne I$ the second deep merit of the scenario lies in the one-to-one correspondence between the geometry of Hilbert space and the characteristics of the EP. In this manner, the behavior of dynamics becomes directly controlled by the characteristics of the EP, i.e., by its algebraic multiplicity *N* and by its geometric multiplicity *K*. In the vicinity of a given EP, the latter two integers will characterize the dynamics in a unified qualitative manner tractable as a certain quantum analogue of the classical Thom’s catastrophe theory.

## Summary

In Introduction we formulated our present project as a transfer of the Thom’s classical concept of catastrophes to quantum theory. We reminded the readers that the geometric nature of the Thom’s theory (in which the stability of a long-time equilibrium of the system in question is mimicked and simulated by the local stability of a local minimum of the so called Lyapunov function *V*(*x*)) cannot easily be transferred to quantum mechanics, i.a., due to the phenomenon of tunneling (see also^9^).

Now, let us add that there still exist multiple parallels between the present considerations and the Thom’s theory. Indeed, in the latter case, a classification of classical catastrophes was achieved via the reduction of arbitrary *V*(*x*)s to its “canonical” form. The resulting bifurcation scenarios were given the intuitively appealing names (like the “fold catastrophe” with “canonical” one-parametric $V(x)=x^3+ax$, or the “cusp catastrophe” with the two-parametric but still one-dimensional $V(x)=x^4+ax^2+bx$, etc^5^).

All this made the classical Thom’s theory popular. On this background we pointed out, in^88^, that many of the standard Lyapunov functions *V*(*x*) could rather easily be reinterpreted as mimicking certain strictly quantum analogues of the classical elementary catastrophes. Indeed, once we decided to define the catastrophes, qualitatively, as the “sudden shifts in behavior arising from small changes in circumstances”^3^, we were immediately able to reinterpret many (i.e., not all!) Lyapunov functions *V*(*x*) as the “benchmark” quantum potentials in Schrödinger Eq. (1) with $H = -\triangle + V(x)$.

The latter idea found its constructive applications even in more dimensions, with $x \in {\mathbb {R}}^d$ at nontrivial $d=2$ in^89^, or at the more realistic $d=3$ in^90^. Nevertheless, the price had to be paid for the strictly shared locality of the benchmark potentials *V*(*x*). This made the fairly close classical-quantum analogy incomplete and, unfortunately, just approximative. Indeed, a key weakness of the approach lied in the nature of the assignment of a suitable EP parameter $g^{(EP)}$ to the corresponding quantum catastrophe. The reason was that in a way motivated by the above-cited Stone theorem, the Hamiltonians were chosen self-adjoint. This implied that Im$(g^{(EP)}) \ne 0$. Thus, the unavoidable presence of a small imaginary components in the parameters made the process of reaching the phase transition non-unitary. In other words, the simulation of the quantum “energy-level-degeneracy” catastrophe (achieved, in our preceding sections, due to the hidden Hermiticity of *H*) would require an analytic continuation. Without such a modification of the model, the “shifts in behavior” would not be “sudden”, and the well known “avoided level mergers” would be experimentally observed. In comparison, our present, EP-related simulation of the energy-level mergers proved more successful, exact and “unavoided” (see also, in this context, the exactly solvable non-Hermitian differential-operator model in^43^).

In any case, a word of warning must be added. The point is that the domain of the realistic physics in which one deals with the concept of the quantum phase transition (for reference, the readers should consult, e.g., the Sachdev’s classical monograph^24^) is, naturally, much larger than its EP-based subdomain as studied and clarified in our present paper. In parallel, the source of optimism concerning the future developments of our present approach could be sought in the possibility of a partial return to the open-system theory. Indeed, in its more ambitious forms one could use, typically, Lindblad operators^14^ or Liouvillians^91–93^. In such a framework, the present classification of some of the EP-based phase-transition processes could also be, in a next-step development, included.

Many open question emerge in such an open-system setting at present. They are mostly connected with the specific, Liouvillean-picture-related phenomena like quantum jumps^91^, in a way moving beyond the limitations characterizing various standard quantum master equation descriptions^94^. In our preceding text we only stressed that when speaking about Hamiltonians with EPs, one usually deals with the information about the environment which is incomplete. Now, let us add that some more sophisticated open-system Hamiltonians might remain Hermitian and, thus, fundamental. In the constructions of this type (see, e.g.,^95,96^) the consistency of the theory is achieved, via introduction of the so called Langevin force, in the Heisenberg picture, i.e., not in the present SP framework.

## Data Availability

No datasets were generated or analysed during the current study.

## References

[1] Messiah A (1961). Quantum Mechanics.

[2] Thom R (1989). Structural Stability and Morphogenesis: An Outline of a General Theory of Models.

[3] https://en.wikipedia.org/wiki/Catastrophe_theory.

[4] Zeeman EC (1977). Catastrophe Theory-Selected Papers 1972–1977.

[5] Arnold VI (1992). Catastrophe Theory.

[6] Poston J, Stewart I (1978). Catastrophe Theory and Its Applications.

[7] Krokidis X, Noury S, Silvi B (1997). Characterization of elementary chemical processes by catastrophe theory. J. Phys. Chem..

[8] O’Dell DHJ (2012). Quantum catastrophes and ergodicity in the dynamics of bosonic Josephson junctions. Phys. Rev. Lett..

[9] Znojil M (2012). Quantum catastrophes: A case study. J. Phys. A Math. Theor..

[10] Goldberg AZ, Al-Qasimi A, Mumford J, O’Dell DHJ (2019). Emergence of singularities from decoherence: Quantum catastrophes. Phys. Rev. A.

[11] Heiss WD, Müller M, Rotter I (1998). Collectivity, phase transitions, and exceptional points in open quantum systems. Phys. Rev. E.

[12] Heiss WD (2004). Exceptional points - their universal occurrence and their physical significance. Czechosl. J. Phys..

[13] Znojil M (2007). A return to observability near exceptional points in a schematic PT-symmetric model. Phys. Lett. B.

[14] Hatano N (2019). Exceptional points of the Lindblad operator of a two-level system. Molec. Phys..

[15] Naghiloo M, Abbasi M, Joglekar YN, Murch KW (2019). Quantum state tomography across the exceptional point in a single dissipative qubit. Nat. Phys..

[16] Miri MA, Alu A (2019). Exceptional points in optics and photonics. Science.

[17] Li SX, Zhang XQ, Xu Q, Liu M, Kang M, Han JG, Zhang WL (2020). Exceptional point in a metal-graphene hybrid metasurface with tunable asymmetric loss. Opt. Express.

[18] Kato T (1966). Perturbation Theory for Linear Operators.

[19] Bender CM, Boettcher S (1998). Real spectra in non-Hermitian Hamiltonians having PT symmetry. Phys. Rev. Lett..

[20] Stone MH (1932). On one-parameter unitary groups in Hilbert space. Ann. Math..

[21] Bender CM (2007). Making sense of nonhermitian Hamiltonians. Rep. Prog. Phys..

[22] Mostafazadeh A (2010). Pseudo-hermitian quantum mechanics. Int. J. Geom. Meth. Mod. Phys..

[23] Özdemir Ş, Rotter S, Nori F, Yang L (2019). Parity-time symmetry and exceptional points in photonics. Nat. Mater..

[24] Sachdev S (1999). Quantum Phase Transitions.

[25] Znojil M (2020). Passage through exceptional point: Case study. Proc. R. Soc. A Math. Phys. Eng. Sci..

[26] Christodoulides D, Yang J-K (2018). Parity-time Symmetry and Its Applications.

[27] Bender, C. M. (ed. with contributions from P. E. Dorey, C. Dunning, A. Fring, D. W. Hook, H. F. Jones, S. Kuzhel, G. Levai, & R. Tateo) *PT Symmetry in Quantum and Classical Physics* (World Scientific, 2018).

[28] Borisov DI, Růžička F, Znojil M (2015). Multiply degenerate exceptional points and quantum phase transitions. Int. J. Theor. Phys..

[29] Znojil M, Borisov DI (2020). Anomalous mechanisms of the loss of observability in non-Hermitian quantum models. Nucl. Phys. B.

[30] Znojil M (2021). Paths of unitary access to exceptional points. J. Phys. Conf. Ser..

[31] Moiseyev N (2011). Nonhermitian Quantum Mechanics.

[32] Znojil M, Semorádová I, Růžička F, Moulla H, Leghrib I (2017). Problem of the coexistence of several nonhermitian observables in PT-symmetric quantum mechanics. Phys. Rev. A.

[33] Krejčiřík D, Lotoreichik V, Znojil M (2018). The minimally anisotropic metric operator in quasi-Hermitian quantum mechanics. Proc. R. Soc. A Math. Phys. Eng. Sci..

[34] Scholtz FG, Geyer HB, Hahne FJW (1992). Quasi-Hermitian operators in quantum mechanics and the variational principle. Ann. Phys..

[35] Mostafazadeh A (2006). Metric operator in pseudo-Hermitian quantum mechanics and the imaginary cubic potential. J. Phys. A Math. Gen..

[36] Znojil M, Geyer HB (2006). Construction of a unique metric in quasi-Hermitian quantum mechanics: Nonexistence of the charge operator in a 2x2 matrix model. Phys. Lett. B.

[37] Krejčiřík D (2008). Calculation of the metric in the Hilbert space of a PT-symmetric model via the spectral theorem. J. Phys. A Math. Theor..

[38] Ju, C.-Y., Miranowicz, A., Minganti, F., Chan, C.-T., Chen, G.-Y. & Nori, F. Flattening the Curve with Einstein’s Quantum Elevator, arXiv:2107.11910.

[39] Bagarello F (2015). Non-Selfadjoint Operators in Quantum Physics: Mathematical Aspects.

[40] Znojil M (2008). On the role of the normalization factors $$\kappa _n$$ and of the pseudo-metric P in crypto-Hermitian quantum models. Symm. Integ. Geom. Methods Appl..

[41] Znojil M (2001). PT symmetric square well. Phys. Lett. A.

[42] Landau LD, Lifshitz EM (1981). Quantum Mechanics: Non-Relativistic Theory.

[43] Znojil M (1999). PT symmetric harmonic oscillators. Phys. Lett. A.

[44] Char BW, Geddes KO, Gonnet GH, Leong BL, Monagan MB, Watt SM (1993). Maple V Language Reference Manual.

[45] Maple 8, permanent licence 2002.0531 issued, on 24-Jan-2002, by Waterloo Maple Inc.

[46] Znojil M (2020). Quantum phase transitions in nonhermitian harmonic oscillator. Sci. Rep..

[47] Günther U, Stefani F, Znojil M (2005). MHD alpha(2)-dynamo, Squire equation and PT-symmetric interpolation between square well and harmonic oscillator. J. Math. Phys..

[48] Joglekar YN, Bagchi B (2012). Competing PT potentials and the re-entrant PT-symmetric phase: A particle in a box. J. Phys. A Math. Theor..

[49] Lévai G, Kovacs J (2019). The finite PT-symmetric square well potential. J. Phys. A Math. Theor..

[50] Arkhipov II, Minganti F, Miranowicz A, Nori F (2021). Generating high-order quantum exceptional points in synthetic dimensions. Phys. Rev. A.

[51] Trefethen LM, Embree M (2005). Spectra and Pseudospectra.

[52] Krejčiřík D, Siegl P, Tater M, Viola J (2015). Pseudospectra in nonhermitian quantum mechanics. J. Math. Phys..

[53] Znojil M (2019). Unitarity corridors to exceptional points. Phys. Rev. A.

[54] Znojil M (2018). Admissible perturbations and false instabilities. Phys. Rev. A.

[55] Znojil M, Lévai G (2001). Spontaneous breakdown of PT symmetry in the solvable square well model. Mod. Phys. Lett. A.

[56] Znojil M (2005). Solvable PT-symmetric model with a tunable interspersion of non-merging levels. J. Math. Phys..

[57] Bagchi B, Bila H, Jakubsky V, Mallik S, Quesne C, Znojil M (2006). PT-symmetric supersymmetry in a solvable short-range model. Int. J. Mod. Phys. A.

[58] Kalvoda T, Štampach F (2020). New family of symmetric orthogonal polynomials and a solvable model of a kinetic spin chain. J. Math. Phys..

[59] Joglekar YN, Scott D, Babbey M, Saxena A (2010). Robust and fragile PT-symmetric phases in a tight-binding chain. Phys. Rev. A.

[60] Znojil M (2011). Discrete quantum square well of the first kind. Phys. Lett. A.

[61] Znojil M (2014). Solvable non-Hermitian discrete square well with closed-form physical inner product. J. Phys. A Math. Theor..

[62] Znojil M (2006). Matching method and exact solvability of discrete PT-symmetric square wells. J. Phys. A Math. Gen..

[63] Langer H, Tretter C (2004). Czechosl. J. Phys..

[64] Fernandez FM, Garcia J (2014). Critical parameters for non-hermitian Hamiltonians. Appl. Math. Comp..

[65] Znojil M (2021). Exceptional points and domains of unitarity for a class of strongly non-Hermitian real-matrix Hamiltonians. J. Math. Phys..

[66] Lei SJ, Bai D, Ren ZZ, Lyu MJ (2021). Finding short-range parity-time phase-transition points with a neural network chain. Phys. Lett..

[67] Buslaev V, Grecchi V (1993). Equivalence of unstable anharmonic oscillators and double wells. J. Phys. A Math. Gen..

[68] https://en.wikipedia.org/wiki/Chebyshev_polynomials.

[69] https://dlmf.nist.gov/18.

[70] Znojil M (2018). Complex symmetric Hamiltonians and exceptional points of order four and five. Phys. Rev. A.

[71] Znojil M (2007). Tridiagonal PT-symmetric N by N Hamiltonians and a fine-tuning of their observability domains in the strongly non-Hermitian regime. J. Phys. A Math. Theor..

[72] Bagarello F, Gargano F, Roccati F (2019). Tridiagonality, supersymmetry and non self-adjoint Hamiltonians. J. Phys. A Math. Theor..

[73] Jones HF (2007). Scattering from localized non-Hermitian potentials. Phys. Rev. D.

[74] Znojil M (2008). Discrete PT-symmetric models of scattering. J. Phys. A Math. Theor..

[75] Znojil M (2009). Scattering theory using smeared non-Hermitian potentials. Phys. Rev. D.

[76] Ambichl P, Makris KG, Ge L, Chong YD, Stone AD, Rotter S (2013). Breaking of PT symmetry in bounded and unbounded scattering systems. Phys. Rev. X.

[77] Longhi S, Della Valle G (2013). Absence of Floquet scattering in oscillating non-Hermitian potential wells. Phys. Rev. A.

[78] Kuzhel S, Znojil M (2017). Non-self-adjoint Schroedinger operators with nonlocal one point interactions. Banach J. Math. Anal..

[79] Znojil M (2020). Unitary unfoldings of Bose-Hubbard exceptional point with and without particle number conservation. Proc. R. Soc. A Math. Phys. Eng. Sci. A.

[80] Graefe EM, Günther U, Korsch HJ, Niederle AE (2008). A non-Hermitian PT symmetric Bose-Hubbard model: Eigenvalue rings from unfolding higherorder exceptional points. J. Phys. A Math. Theor..

[81] Znojil M (2021). Quantum phase transitions mediated by clustered non-Hermitian degeneracies. Phys. Rev. E.

[82] Hiller M, Kottos T, Ossipov A (2006). Bifurcations in resonance widths of an open Bose-Hubbard dimer. Phys. Rev. A.

[83] Jin L, Song Z (2013). Scaling behavior and phase diagram of a PT-symmetric non-Hermitian Bose-Hubbard system. Ann. Phys..

[84] Znojil M (2021). Bose-Einstein condensation processes with nontrivial geometric multiplicites realized via PT-symmetric and exactly solvable linear-Bose-Hubbard building blocks. Quantum Rep..

[85] Sloane, N. J. A. Number of partitions of n that do not contain 1 as a part. http://oeis.org/A002865/ (Accessed 30 Oct 2020).

[86] Hassan AU, Hodaei H, Miri MA, Khajavikhan M, Christodoulides DN (2019). Nonlinear reversal of the PT-symmetric phase transition in a system of coupled semiconductor microring resonators. Phys. Rev. A.

[87] Konotop VV, Yang JK, Zezyulin DA (2016). Nonlinear waves in PT-symmetric systems. Rev. Mod. Phys..

[88] Znojil M (2020). Arnold’s potentials and quantum catastrophes. Ann. Phys..

[89] Znojil M (2020). Relocalization switch in a triple quantum dot molecule in 2D. Mod. Phys. Lett. B.

[90] Znojil M (2020). Polynomial potentials and coupled quantum dots in two and three dimensions. Ann. Phys..

[91] Minganti F, Miranowicz A, Chhajlany RW, Nori F (2019). Quantum exceptional points of non-Hermitian Hamiltonians and Liouvillians: The effects of quantum jumps. Phys. Rev. A.

[92] Minganti F, Miranowicz A, Chhajlany RW, Arkhipov II, Nori F (2020). Hybrid-Liouvillian formalism connecting exceptional points of non-Hermitian Hamiltonians and Liouvillians via postselection of quantum trajectories. Phys. Rev. A.

[93] Chen, W.-J., Abbasi, M., Ha, B., Erdamar, S., Joglekar, Y. N. & Murch, K. W. Decoherence Induced Exceptional Points in a Dissipative Superconducting Qubit, arXiv:2111.04754. 10.1103/PhysRevLett.128.110402 35363025

[94] Purkayastha, A. The Lyapunov equation in open quantum systems and non-Hermitian physics. http://arxiv.org/abs/2201.00677.

[95] Miri MA, Alù A (2016). Nonlinearity-induced PT-symmetry without material gain. N. J. Phys..

[96] Wang Y-X, Clerk AA (2019). Non-Hermitian dynamics without dissipation in quantum systems. Phys. Rev. A.

